# Massive mixed epithelial-stromal tumour of seminal vesicle requiring challenging abdominoperineal resection: a case report and review of literature

**DOI:** 10.1093/jscr/rjad490

**Published:** 2023-08-29

**Authors:** Zirong Yu, Kay Tai Choy, Ferdinand Ong, Evan Williams, Sanjeev Naidu, Bernard M Smithers, Anjan Gurung, Nicholas Lutton

**Affiliations:** Department of General Surgery at Princess Alexandra Hospital-199 Ipswich Rd, Woolloongabba, QLD 4102, Australia; Department of General Surgery at Princess Alexandra Hospital-199 Ipswich Rd, Woolloongabba, QLD 4102, Australia; Department of General Surgery at Princess Alexandra Hospital-199 Ipswich Rd, Woolloongabba, QLD 4102, Australia; Department of General Surgery at Princess Alexandra Hospital-199 Ipswich Rd, Woolloongabba, QLD 4102, Australia; Department of General Surgery at Princess Alexandra Hospital-199 Ipswich Rd, Woolloongabba, QLD 4102, Australia; Department of General Surgery at Princess Alexandra Hospital-199 Ipswich Rd, Woolloongabba, QLD 4102, Australia; Department of General Surgery at Princess Alexandra Hospital-199 Ipswich Rd, Woolloongabba, QLD 4102, Australia; Department of Anatomical Pathology at Princess Alexandra Hospital-199 Ipswich Rd, Woolloongabba, QLD 4102, Australia; Department of General Surgery at Princess Alexandra Hospital-199 Ipswich Rd, Woolloongabba, QLD 4102, Australia

**Keywords:** mixed epithelial-stromal tumours, massive, seminal vesicle, tumour, resection

## Abstract

Mixed epithelial-stromal tumours (MESTs) are a rare biphasic tumour that frequently arise in women from the renal and urogenital tract. They are also seen in men but are exceptionally uncommon with only few cases reported to originate from the seminal vesicles. Malignant transformation of its epithelial or stromal components is possible; however, by in large, these tumours are benign in nature. We report the case of a 48-year-old man with no remarkable medical or surgical history who presented with a huge expanding pelvic and intra-abdominal mass that required extensive surgical management including a pelvic exenteration. Histopathological analysis concluded the diagnosis of benign MEST originating from the seminal vesicles with no malignant features. No further systemic therapy was recommended for our patient. Given the technical intricacy in the operative resection of this tumour, we aim to present our findings and surgical management of this complex MEST.

## Introduction

Mixed epithelial-stromal tumours (MESTs) are a rare and mostly benign neoplasm that arise commonly from the urogenital tract [1]. MESTs are often diagnosed in perimenopausal women and can vary in size, with literature describing ranges between 2.5 and 29 cm [2]. The pathogenesis of MEST of seminal vesicles is unknown; however, there has been growing evidence of its relationship with progesterone and oestrogen exposure as well as a genetic basis for its disease [3]. Here, we report a case of MEST in a middle-aged man with a significant tumour burden in his pelvic and abdominal cavity, requiring extensive surgical resection through intraperitoneal, extraperitoneal, and perineal approaches to achieve clear margins via pelvic exenteration.

## Case report

**Figure 1:**
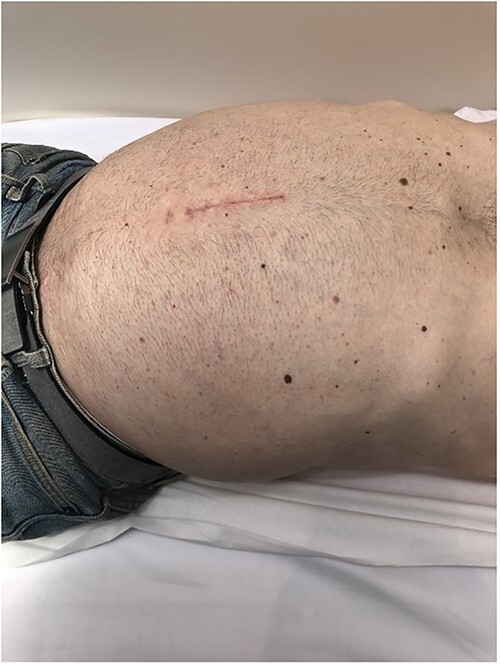
Pre-operative examination.

A 48-year-old man with 6 months of worsening abdominal distension was referred with a diagnostic abdominal Computed Tomography (CT) scan revealing a large mass in his abdomen and pelvis. He reported no urinary or faecal obstructive symptoms, vomiting, loss of appetite, or any positional or exertional dyspnoea. There was no relevant personal or familial medial history and no previous abdominal surgeries. On examination, his abdomen was markedly distended (Fig. 1) with a palpable non-tender soft mass extending from the xiphoid process to the pubic symphysis.

**Figure 2:**
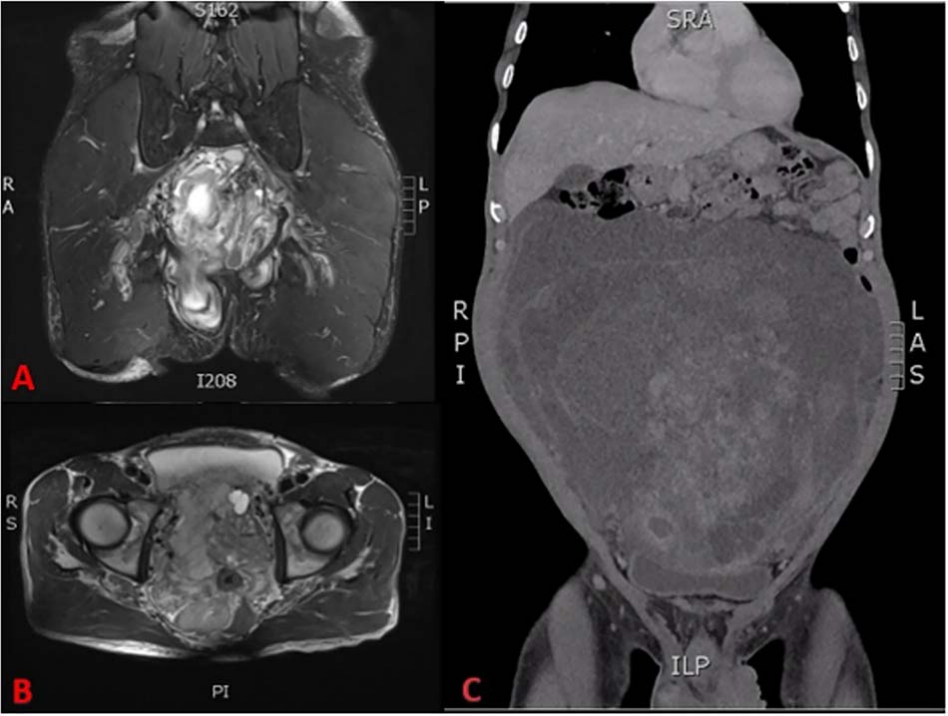
(A) T2 coronal section of MRI pelvis showing tumour invasion of the pelvic floor, (B) T2 axial MRI pelvis showing tumour involvement of the left seminal vesicle and, (C) coronal CT scan showing compression of the bladder and tumour burden.

His full blood count, biochemistry, inflammatory, and tumour markers were within the normal range. The CT scan revealed a large pelvic extraperitoneal mass displacing bowels with heterogenous enhancing solid and non-enhancing cystic components (Fig. 2C). MRI evaluation of the pelvis showed a heterogenous and hyperintense lesion with internal septation and cystic changes invading the pelvic floor (Fig. 2A, B).

An incisional biopsy was performed via a mini-laparotomy, which reported an initial diagnosis of angiomyofibroblastoma.

**Figure 3:**
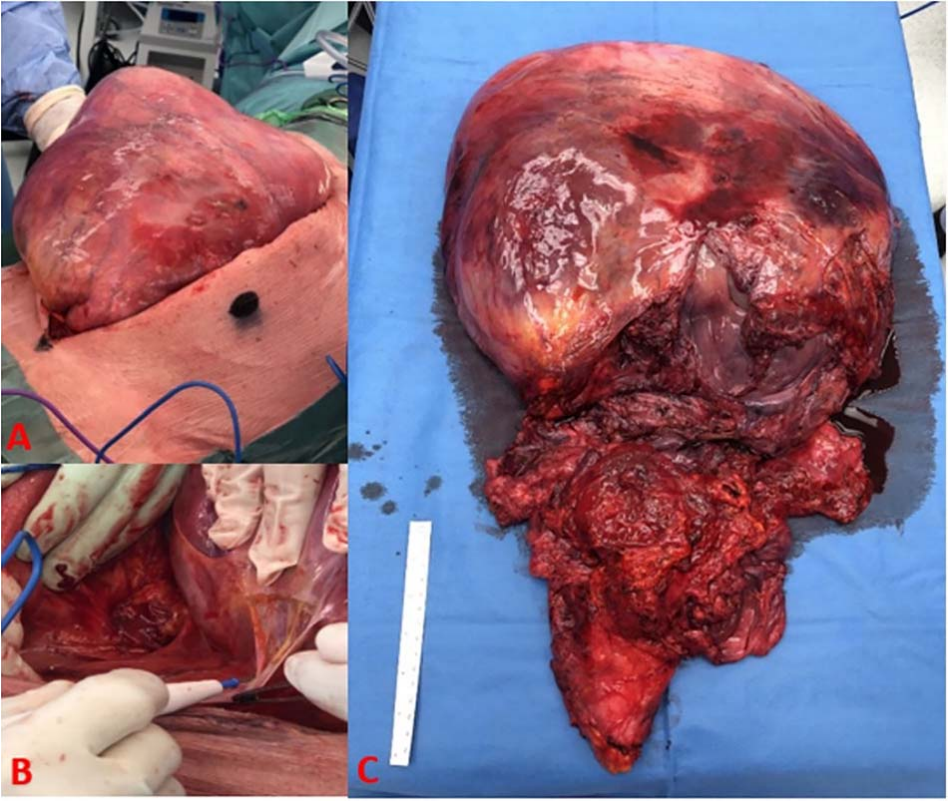
(A) Tumour burden on initial incision, (B) dense adhesive connection of tumour to retroperitoneal structures, and (C) complete excision of tumour with bladder, prostate, rectum, and seminal vesicle.

The patient underwent an elective laparotomy, which showcased a very large abdominal mass (Fig. 3A). Initial intra-operative examination revealed a smooth, well encapsulated mass that mobilized up in the upper abdomen. However, in the lower abdomen, intraperitoneal and pelvic dissection of the tumour was hampered by dense adherence to the rectum, bladder, and distal ureters with obliteration of dissection planes. Thus, an extraperitoneal approach to the pelvic viscera was commenced but the gross involvement of the bladder musculature along with the need to take the pelvic visceral vessels mandated a cysto-prostatectomy. Due to extensive disruption by tumour of the pelvic floor and the rectal involvement, there was little possibility for restoration of gastrointestinal continuity hence an abdominoperineal excision of the rectum was performed to achieve complete tumour-free resection. An ileal conduit was constructed, and end colostomy was performed.

The resected tumour measured 65 cm × 36 cm × 9 cm and weighed 8.08 kg. There was a well demarcated outer capsule (Fig. 3C). The tumour surface was smooth, homogenous with observable extensive capillary networks and contained no signs of infection or necrosis.

**Figure 4:**
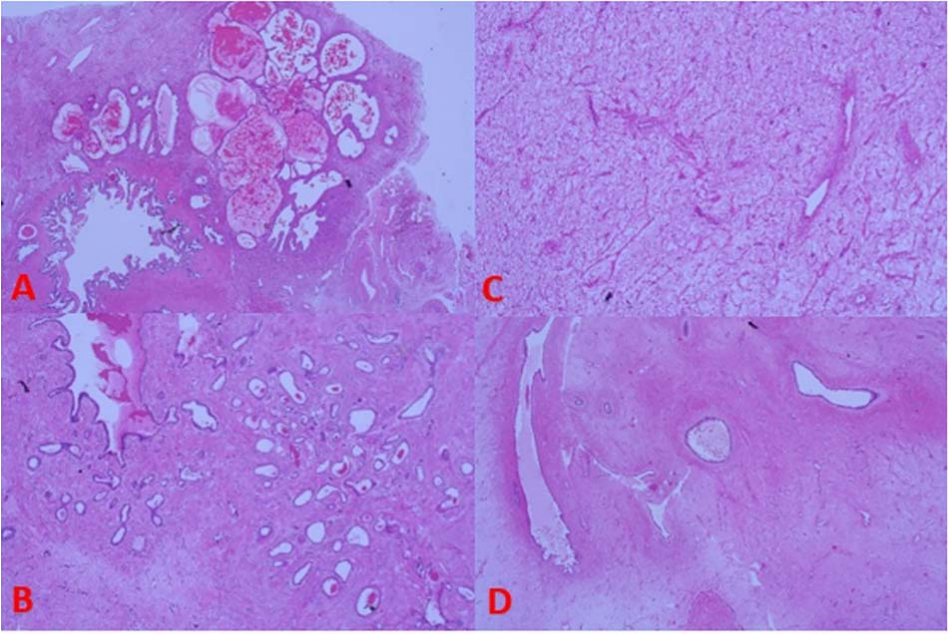
(A) Tumour histology next to seminal vesicle, (B) epithelial glandular component, (C) stromal component, and (D) phyllodes-like pattern on histology.

Histopathology reported a biphasic tumour comprised an epithelial surface (Fig. 4B) with cystic and glandular structures and no signs of atypia along with a stromal component (Fig. 4C) containing uniform spindle cells. Discussions with the examining pathologist favoured the tumour to have originated from the seminal vesicles given its composition and proximity (Fig. 4A). A phyllodes-like pattern was also seen (Fig. 4D).

Immunohistochemistry of the stroma showed positive staining for oestrogen, progesterone, desmin, and smooth muscle actin. The glandular component was cytokeratin positive with intact basal cells and stained negative for prostate markers (Prostate-Specific Antigen, Prostate Specific Acid Phosphatase, NKX3.1).

Post-surgery the patient remained well and was discharged 2 weeks later with planned radiological follow-up.

## Discussion

MESTs are a rare soft-tissue tumour that commonly arise from the urogenital tract with biological behaviours ranging from benign to malignant [4].

MEST typically affects perimenopausal women with a preference to involve renal parenchyma. In males, it is exceptionally rare and is associated with a history of hormonal therapy (androgen deprivation/oestrogen therapy) often coupled with managing prostate cancer [5].

MEST occurrence in the SV is very rare. A recent systematic review by Babar *et al.* [6] reported only 36 cases of MEST SV in the English literature with varying presenting complaints ranging from asymptomatic, urinary retention, haematuria, and abdominal pain. MESTs were originally described in 1944 by Plautt *et al.* [7] who reported a large 15 cm abdominal mass originating from the seminal vesicles and initially termed this cystomyoma, due to the biphasic epithelial and stromal components. Subsequent reports included different nomenclatures such as cystadenoma, papillary cystadenoma, mullerian adenosarcoma-like tumour, Phyllodes tumour, and benign fibroepithelial tumour [1, 8]. Complementing a recent review by Reikie *et al.* [9], the World Health Organisation has reported clear criteria for diagnosis of MEST [10] proposing a common terminology (MEST), to designate the tumours of the seminal vesicle that contain both epithelial and stromal components.

Malignant MESTs have been described using a three-tiered grading system (low/intermediate/high) based on stromal atypia, mitotic activity, pleomorphism, and necrosis [11, 12]. Within the literature, up to 94% of all MESTs of SV are low-grade, and, regardless of size, there are no reports of metastasis and although local recurrence is possible, these tumours have generally good prognosis [6]. Malignant transformation of MEST is possible and indicates poorer outcomes [13].

MEST diagnosis heavily depends on histopathology as tumours of the pelvic/urogenital tract can have multiple aetiologies and, even with sophisticated imaging modalities, clear differentiation can be difficult [4, 14].

Interestingly, our initial biopsy of the tumour was favoured to represent a myofibroblastic lesion such as an angiomyofibroblastoma or cellular angiofibroma due to the biopsy only containing stromal component. However, upon reviewing the entire resected specimen, both stromal and epithelial components were present, which fits well with MEST of SV.

Differentiating benign from malignant MEST is important, with the former commonly exhibiting stromal blandness and the absence of mitotic activity [12]. Histologically, all MESTs contain hypercellular stroma consisting of spindle cells interspersed between glands with immunohistochemical staining revealing diffuse stromal positivity for SMA, desmin, h-caldesmon, oestrogen, and progesterone [2, 4]. More worrisome pathology, such as infiltrative borders, cell atypia, increased mitotic activity, haemorrhage, and necrosis, are associated with malignant potential [9].

Therapeutic management is centred on complete surgical resection of tumour-burdened viscera with a systematic review describing resection using open (57%), laparoscopic (26%), or robotic (17%) techniques [6]. Previous case reports have described intraperitoneal excision of smaller tumours laparoscopically [4, 15].

However, the extensive size and involvement of pelvic organs, as seen in our case, has not been reported and highlights the need for adequate imaging and preparation for extensive pelvic organ dissection, and where required, as we found, resection of those organs to obtain a complete resection of the tumour.

## Conclusion

We report a very rare benign tumour of the seminal vesicles that presented with a massive abdominal component emanating from the locally advanced pelvic component. To achieve complete resection, the man required a pelvic exenteration. The prognosis from this extensive benign tumour should be excellent.

## Data Availability

The data that support the findings of this study are available from the corresponding author, ZY, upon reasonable request.
